# Impact of a brief peri-operative counselling session on parental awareness of passive smoking in paediatric ambulatory surgery: A single-centre observational study

**DOI:** 10.1097/EA9.0000000000000121

**Published:** 2026-05-22

**Authors:** Claude Hallet, Adeline Prijs, Loïc Le Jeune, Florian Beck, Vincent L. Bonhomme, Michele Carella

**Affiliations:** From the Department of Anesthesia and Intensive Care Medicine, Liege University Hospital (CH, AP, FB, VLB, MC), Inflammation and Enhanced Rehabilitation Laboratory (Regional Anesthesia and Analgesia), GIGA-I3 Thematic Unit, GIGA-Research, Liege University (MC), Nursing Department, Liège University Hospital (LLJ) and Anesthesia and Peri-operative Neuroscience Laboratory, GIGA-Consciousness, GIGA-Neuroscience, Liege University, Liege, Belgium (FB, VLB)

## Abstract

**BACKGROUND:**

Exposure to second-hand smoke (SHS) from parents increases paediatric peri-operative respiratory risk, yet peri-operative screening and counselling are rarely implemented.

**OBJECTIVE:**

To assess whether a single brief counselling session delivered during the paediatric ambulatory surgery pathway increases parental awareness of SHS harms and influences short-term self-reported behaviour.

**DESIGN:**

Prospective observational before–after study with 30-day follow-up.

**SETTING:**

Single-centre tertiary-level university hospital ambulatory surgery unit, Liège, Belgium; data collected from 7 November 2024 to 27 March 2025.

**PATIENTS:**

Parents who self-reported active tobacco use and whose child was scheduled for elective paediatric ambulatory surgery at the University Hospital of Liège, Belgium. Of 43 eligible dyads, 31 parents consented and completed baseline and immediate postintervention questionnaires; 26 completed 30-day follow-up.

**INTERVENTION:**

One standardised ~15-min counselling session by a certified tobacco counsellor covering types of smoke exposure, health and anaesthetic risks and practical measures to reduce household exposure.

**MAIN OUTCOME MEASURES:**

Primary outcome was change in a summed awareness score (six items, 0 to 10 each; total 0 to 60) from precounselling to immediate postcounselling. Secondary outcomes included item-level changes, session acceptability and self-reported behavioural change at 30 days.

**RESULTS:**

A brief peri-operative counselling session markedly improved parental awareness of SHS. At 30 days, self-reported behavioural change remained modest. The awareness score was exploratory and not formally validated. Randomised studies with objective exposure measures and longer follow-up are needed.

**CONCLUSION:**

A single brief peri-operative counselling session improved parental awareness of SHS harms, with limited short-term impact on self-reported behaviour.

**TRIAL REGISTRATION:**

NCT07144982. Approved by the Comité d’Ethique Hospitalo-Facultaire Universitaire de Liège; President: Professor D. Ledoux; IRB number: 707 (Belgian ref. B7072024000085; internal ref. 2024/327).

**KEY POINTS**

- Parental exposure to second-hand smoke increases paediatric peri-operative respiratory risk and is frequently unaddressed during preoperative evaluation.
- A single brief, standardised counselling session delivered during the paediatric ambulatory pathway significantly increased parental awareness of SHS harms.
- The largest item-level gains concerned perceived anaesthetic risk and misconceptions about tertiary/indirect exposure.
- Session acceptability was high (median satisfaction 10/10), but self-reported behavioural change at 30 days was modest (median 2.5/10).
- While the peri-operative consultation is a feasible ‘teachable moment’ to raise awareness, sustained behaviour change likely requires multicomponent follow-up (visual aids, objective exposure measures and structured cessation support) and warrants evaluation in controlled trials.

## Introduction

Second-hand smoke (SHS) – the involuntary inhalation of tobacco smoke present in the environment – is a major public-health concern, particularly for children.^1^ In Belgium and elsewhere, public health bodies have long highlighted the need for systematic management of passive smoking in paediatric practice, calling on healthcare professionals to recognise and address its harmful effects.^2^ Roughly 40% of children worldwide are regularly exposed to SHS, with well documented short-term and medium-term consequences including recurrent respiratory infections, new-onset or worsened asthma and increased risk of chronic cardiometabolic and pulmonary disease^3–5^; neurodevelopmental and behavioural problems have also been linked to early exposure.^6,7^ The physiologic immaturity of the respiratory and immune systems, coupled with children's typical proximity to adults, makes them vulnerable to inhaled toxins.^6–8^

This vulnerability is especially relevant during the peri-operative period. Observational data and meta-analyses indicate a clear association between environmental tobacco smoke and an increased risk of adverse respiratory events under general anaesthesia in children – laryngospasm, bronchospasm, coughing and intra-operative desaturation being among the most frequent.^6^ In the ‘Incidence of severe critical events in paediatric anaesthesia (APRICOT)’ multinational observational study, peri-operative respiratory complications occurred in approximately 3.1% of paediatric anaesthetic procedures, with passive smoke exposure recognised as a contributory risk factor.^9,10^ Despite this evidence, passive smoking remains infrequently assessed or targeted during routine pre-anaesthetic evaluation, and peri-operative strategies to detect and mitigate household tobacco exposure are not well established.^11^

Some practical questions therefore remain unanswered: how best identify at-risk children prior to surgery, which peri-operative adaptations (respiratory optimisation, intensified postoperative monitoring) to reduce risk, and crucially, what is the temporal relation between parental smoking cessation and improvement in the child's peri-operative morbidity. The pre-anaesthetic consultation represents a timely, teachable moment when parents may be particularly receptive to health messages and when peri-operative care pathways can be tailored to reduce risk.^12^

On this basis, we designed a preventive educational intervention delivered during the preoperative anaesthesia consultation. The present study evaluates whether a brief information and counselling session improves parental awareness of the harms of passive smoke exposure in the context of paediatric ambulatory surgery, and whether this change in awareness translates into intentions to modify household smoking behaviours. By embedding tobacco exposure counselling within standard peri-operative pathways, we aim to explore a feasible approach to reduce children's peri-operative risk and promote long-term protective changes in the home environment.

## Methods

This observational, noninterventional study was approved by our local Institutional Review Board (Comité d’Ethique Hospitalo-Facultaire Universitaire de Liège; President: PIRB number: 707; Belgian reference B7072024000085; internal reference 2024/327). The study evaluated the immediate-term and short-term impact of a brief, structured counselling session administered by a certified tobacco-cessation counsellor on parental awareness of passive smoking risks in the setting of paediatric ambulatory surgery. The Institutional Review Board determined that the project constituted noninterventional/observational research and therefore did not require registration in a clinical trials registry. Although prospective registration is not mandatory for observational studies, the protocol was registered after completion of data collection but prior to any data analysis to enhance transparency and minimise concerns regarding selective reporting; the registration record is available at NCT07144982 (https://clinicaltrials.gov/study/NCT07144982). Informed consent was obtained from all participants. The study was conducted in accordance with the Declaration of Helsinki and follows the STrengthening the Reporting of OBservational studies in Epidemiology (STROBE) guidelines for observational cohort research.

### Study setting and population

Parents who self-reported active tobacco use and whose child was scheduled for elective paediatric ambulatory surgery were eligible. Potential participants were identified from the operating schedule and the pre-anaesthetic consultation notes. All consecutive parents who met the predefined inclusion criteria during the study period were approached for participation; those who agreed provided written informed consent and were enrolled. Approximately 1 week before the scheduled procedure, parents were contacted by telephone, informed about the study objectives and procedures and invited to participate; written informed consent was obtained prior to inclusion. Recruitment and data collection occurred during the child's ambulatory stay.

Each enrolled parent received a single, face-to-face education/counselling session delivered by a certified tobacco counsellor while present in the ambulatory surgery unit. The session followed a standardised script delivered by the same counsellor for all participants to ensure consistency of content; however, formal fidelity assessment (e.g. checklist scoring or independent observation) was not performed in this exploratory study. If both parents of the same child were active smokers, each could be included as a separate parent–child dyad. Given the exploratory design and limited sample size, clustering at the household level was not modelled. The session lasted approximately 15 min and followed a standardised script (see Appendix). Core components were: explanation of types of tobacco smoke exposure (primary, secondary and tertiary smoke) and their relevance in enclosed environments (home, car); definition and health consequences of passive smoking for children, with emphasis on peri-operative respiratory risks; practical measures to reduce exposure (smoke-free home/car rules, behavioural tips) and brief guidance on initial smoking-cessation steps and referral options. The counselling was delivered using a structured script with short, targeted information modules supported by simple visual aids and take-home recommendations (e.g. smoke-free home and car rules). No formal reminder or reinforcement session was scheduled as part of this exploratory design, although parents were offered referral to existing tobacco-cessation services if interested.

### Recorded parameters and outcomes

For each parent, we recorded age, sex, educational level, tobacco type and intensity (number of cigarettes per day), and the child's exposure pattern (smoking inside in presence of the child, inside in the child's absence, or exclusively outdoors). Data on nicotine dependence scores or previous quit attempts were not collected in this exploratory study. Child data included age, weight, height, relevant medical history, type of surgery [ear, nose and throat (ENT) vs. non-ENT], occurrence of peri-operative respiratory complications and airway management modality (endotracheal tube or laryngeal mask).

The primary outcome was the change in parental awareness of passive-smoking harms, as measured by a six-item questionnaire administered on a 0 to 10 numeric rating scale (0 = strongly disagree to 10 = strongly agree). For questions 3, 4 and 5, the scoring direction was inverted, with 0 indicating ‘strongly agree’ and 10 indicating ‘strongly disagree’. The questionnaire items probed perceived anaesthetic risk, general health risk to the child and beliefs about the safety of smoking outside or at distance from the child, as well as intention to reduce exposure. The questionnaire was developed by the study team based on previously published tools assessing parental perceptions of SHS exposure, reviewed by tobacco-cessation specialists, and pilot-tested for clarity; however, it was not formally validated. Scores for the six items were summed to generate a total knowledge/awareness score (possible range 0 to 60). The summed awareness score reflects perceived knowledge and attitudes and was not intended to measure actual SHS exposure reduction or clinical peri-operative outcomes. The items were: (1) ‘I consider that my smoking habits increase the anaesthetic risk for my child’; (2) ‘I consider that my smoking habits represent an increased health risk for my child’; (3) ‘I think that smoking outdoors protects my child from second-hand smoke exposure’; (4) ‘I think that smoking indoors in the absence of my child does not expose them to second-hand smoke’; (5) ‘I think that smoking indoors in the presence of my child, but at a sufficient distance, does not expose them to second-hand smoke’; and (6) ‘I intend to increase my efforts to reduce my children's exposure to second-hand smoke’.

Filling of the questionnaire occurred three times: at baseline on arrival to the ambulatory unit (precounselling, T0); immediately after the counselling session, during the child's procedure and prior to discharge (postcounselling, T1) and by telephone at 30 days postintervention (T30) to assess reported behavioural change and persistence of attitudes. Acceptability was assessed using a single, locally developed, nonvalidated numeric rating question administered at the end of the session, evaluating participants’ perceived usefulness, clarity, accessibility and completeness of the counselling.

### Statistical analysis

Sample size was calculated from unpublished pilot data (*n* = 20) showing a mean total score of 42 +/− 9. Assuming a 15% improvement in the total score after counselling, corresponding to a large effect size (Cohen's *d* ≈ 0.70), *α* = 0.05 and power = 90%, the required sample was 32 participants after allowance for an anticipated 15% dropout. Normality of distributions was assessed by the skewness and the Shapiro–Wilk test. For paired comparisons (T0 vs. T1). we used the paired Student's *t* test for normally distributed outcomes and the Wilcoxon signed-rank test for nonparametric paired data. Item-level comparisons of the six questionnaire items were considered exploratory secondary analyses, and no formal adjustment for multiplicity was applied. A two-tailed *P* less than 0.05 was considered statistically significant. No imputation was performed; analyses were based on complete cases (T0 to T1: participants with both questionnaires; T30 analyses restricted to participants with available follow-up). All statistical analyses were performed using R software (version 4.3.2; R Foundation for Statistical Computing, Vienna, Austria).

## Results

**Fig. 1:**
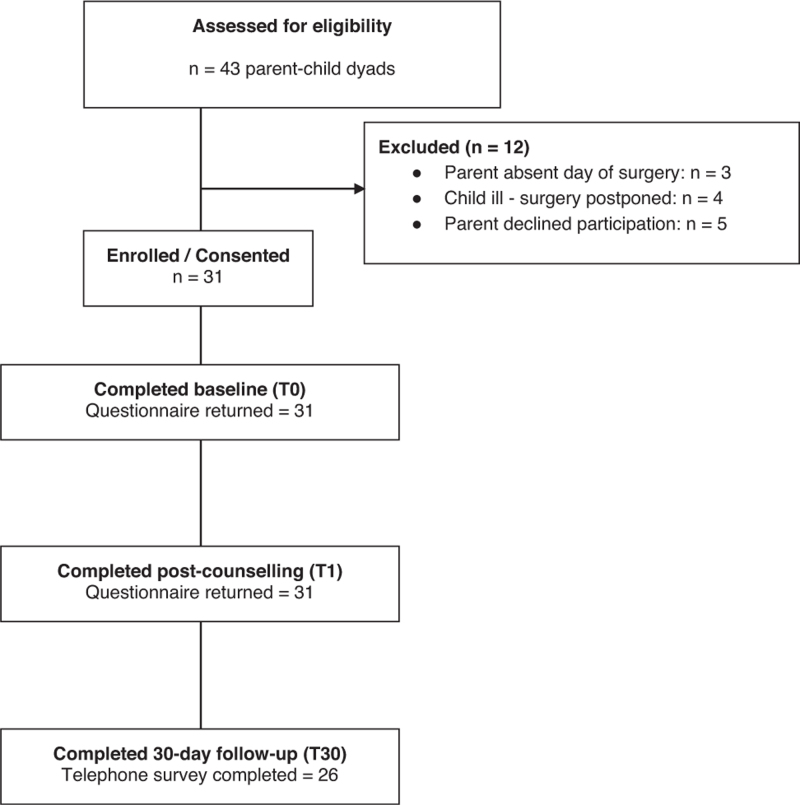
STROBE flow diagram of participant recruitment and follow-up. STROBE, STrengthening the Reporting of OBservational studies in Epidemiology.

Data were collected between 7 November 2024 and 27 March 2025 at the University Hospital of Liège, Belgium. Of 43 potentially eligible dyads approached, 31 agreed to participate and completed all study procedures; no questionnaires were missing, and therefore all 31 participants were included in the analyses (Fig. 1). Among the 12 dyads not included, three parents were absent on the day of the child's surgery, four procedures were postponed because the child was ill and five parents declined participation. Only three households included both parents, representing a small proportion of the sample.

The children comprised a young, relatively homogeneous group with a median [IQR] age of 5 years [3 to 8], median weight 20 kg [16 to 28] and mean height 116 cm (±24.1). Two children (6.2%) had experienced an upper respiratory tract infection during the month preceding surgery. Surgical procedures were evenly distributed between ENT (*n* = 16; 51.6%) and non-ENT specialties (*n* = 15; 48.4%). No peri-operative respiratory complications were observed.

Parents’ mean age was 37.9 years (±5.8) and 18/31 (58.1%) were women. Educational level was predominantly secondary (20/31; 64.5%), with 10/31 (32.3%) having nonuniversity higher education and 1/31 (3.2%) university-level education. Regarding smoking behaviour, 21/31 parents (67.7%) reported smoking exclusively outdoors, 9/31 (29.0%) reported smoking indoors only when the child was absent, and 1/31 (3.1%) smoked indoors in the child's presence. Self-reported tobacco consumption ranged from approximately 10 to 30 cigarettes per day (≈0.5 to 1.5 packs per day).

Acceptability of the counselling session was high: overall satisfaction had a median of 10/10 [8 to 10]. Behavioural change reported at 30 days was modest, with a median self-reported change of 2.5/10 [0 to 8] based on 26 respondents who completed the day-30 telephone follow-up.

Knowledge and attitude scores (six items, summed to a 0 to 60 total) increased significantly following the counselling session from T0 to T1. The median total score rose from 34 [27.5 to 41.5] precounselling to 46 [33 to 50] postcounselling (Wilcoxon signed-rank test, *P* = 0.0026). Item-level analyses showed significant improvements for two items: Question 1 (‘I consider that my smoking habits increase anaesthetic risk for my child’) increased from a median of 5.0 [0.5 to 6.0] to 8.0 [3 to 10] (*P* = 0.0017), and Question 4 (‘I think smoking indoors in the absence of my child does not expose them to second-hand smoke’) increased from 8 [2.5 to 10] to 10 [7 to 10] (*P* = 0.012). The remaining items did not show statistically significant changes. Item-level data for all six components of the summed awareness score are detailed in Table 1.

**Table 1:** Parental awareness and attitudes toward second-hand smoke: questionnaire items (0 to 10 numeric rating scale)

	T0	T1	*P* value
1. I consider that my smoking habits increase the anaesthetic risk for my child (NRS)	5 [0.5 to 6]	8 [3 to 10]	0.0017
2. I consider that my smoking habits represent an increased health risk for my child (NRS)	5 [4 to 8.5]	8 [3 to 10]	0.14
3. I think that smoking outdoors protects my child from second-hand smoke exposure (NRS)	1 [0 to 3]	1 [0 to 5]	0.23
4. I think that smoking indoors in the absence of my child does not expose them to second-hand smoke (NRS)	8 [2.5 to 10]	10 [7 to 10]	0.012
5. I think that smoking indoors in the presence of my child, but at a sufficient distance, does not expose them to second-hand smoke (NRS)	10 [6.5 to 10]	10 [8.5 to 10]	0.058
6. I intend to increase my efforts to reduce my children's exposure to second-hand smoke (NRS)	10 [8 to 10]	10 [8.5 to 10]	0.35

Data are presented as median with interquartile range [IQR]. NRS, Numeric Rating Scale; T0, baseline assessment on arrival to the ambulatory unit (precounselling); T1, immediate postcounselling assessment prior to discharge.

## Discussion

This study demonstrates that a single, brief counselling session delivered by a certified tobacco counsellor during the paediatric peri-operative pathway was associated with a measurable and immediate increase in parental awareness of the harms of passive smoking. The statistically significant rise in the overall knowledge score – and particularly the improvements for items probing perceived anaesthetic risk (Q1) and misconceptions regarding tertiary smoke (Q4) – suggests that a focused, context-specific message can be effectively received by parents at a time of heightened peri-operative attention. These findings are consistent with prior reports showing that targeted peri-school or school-based interventions combining motivational interviewing and objective feedback can modify parental perceptions and, to some extent, behaviours.^12–14^ Unlike conventional smoking-cessation programmes that rely on repeated sessions or dedicated clinics, our intervention was intentionally designed as a brief (~15 min), highly pragmatic peri-operative counselling encounter delivering concise, targeted SHS messages within the ambulatory surgery pathway, aiming to maximise feasibility, accessibility and uptake in a real-world setting. However, given the single-arm observational design, these findings should be interpreted cautiously and considered exploratory, representing proof-of-concept data to inform the design of adequately powered multicentre randomised studies evaluating structured peri-operative counselling pathways. Increased awareness should not be interpreted as evidence of reduced SHS exposure or improved peri-operative safety; objective exposure measures and clinical endpoints were not assessed in this exploratory study.

The differential item responses provide useful insight into the mechanisms of change. Q1 improved strongly, suggesting that situating the message within the emotionally salient peri-operative context helps parents link smoking to immediate, defined risks for their child – an effect that may be harder to achieve in less salient settings. These interpretations are hypothetical and were not directly assessed in this study; no mechanistic behavioural measures were collected. Q4's improvement shows that education reduced misconceptions about tertiary exposure: parents were more likely after counselling to recognise that the absence of a child at the moment of smoking does not fully protect them from residual contaminants. By contrast, beliefs that are culturally entrenched – such as the protective value of smoking outdoors (Q3) or the broad, abstract notion that parental smoking harms long-term child health (Q2) – were less amenable to change after one brief session. The near-significant trend for Q5 (distance protects) further suggests that some commonly held rationalisations are beginning to be questioned but likely require reinforcement through multimodal evidence (visual demonstrations, objective measures) to be overturned. High baseline scores for some items suggest a possible ceiling effect, which may have reduced the sensitivity of the questionnaire to detect further improvement in these domains and should be considered when interpreting nonsignificant item-level results.

Importantly, intention to change behaviour (Q6) did not significantly increase. This gap between improved knowledge and limited short-term behavioural change is neither unexpected nor unique. Translation of awareness into action is influenced by multiple factors, including readiness to change, nicotine dependence, socioeconomic context and access to cessation support, and usually requires repeated contact, practical tools and ongoing reinforcement. Previous studies similarly show that single-point interventions may increase awareness, whereas sustained smoking cessation or meaningful reduction in household exposure typically requires structured follow-up and dedicated support.^7,15,16^ Overall, these findings illustrate the well described gap between knowledge acquisition and durable behavioural change, underscoring the need for repeated counselling, objective feedback and integrated cessation pathways. A single brief (~15 min) counselling session is unlikely to overcome the complex barriers to reducing household SHS exposure, including nicotine dependence, readiness to change, socio-economic constraints and access to cessation support, which typically require repeated interventions and structured follow-up.

Several limitations should be acknowledged. First, the awareness score was specifically constructed for this study and has not undergone formal psychometric validation; its reliability and external validity therefore need confirmation in larger multicentre cohorts. Second, the sample size was small (*n* = 31), limiting statistical power for item-level and subgroup analyses (e.g. by educational level or baseline smoking intensity). Although the study was adequately powered for the primary endpoint, it was not powered for these exploratory comparisons, and multiple testing increases the risk of type I error; item-level findings should therefore be interpreted cautiously and considered hypothesis-generating pending confirmation in larger cohorts. Attrition at 30-day follow-up was modest, but participants lost to follow-up were not formally compared with completers, which may introduce attrition bias. Third, because this was a single-arm pre/post observational study without a control group, causal inference is limited and the observed changes cannot be definitively attributed to the intervention; secular trends, the emotional context of imminent surgery, or social desirability bias may also have contributed, and randomised or cluster-randomised trials are needed to confirm these findings. In addition, in a small number of cases, both parents of the same child were enrolled, and the analysis did not account for potential clustering at the household level, which should be addressed in future larger studies. Fourth, outcomes relied on self-reported awareness and behavioural change, which are inherently susceptible to reporting and social desirability bias; although appropriate for this exploratory, participant-centred study, future research should incorporate objective exposure biomarkers (e.g. cotinine or environmental particulate measurements) to strengthen validity. Information on nicotine dependence severity and prior cessation attempts was not available and may represent residual confounding. Fifth, follow-up was short (1 month), limiting conclusions about persistence of awareness gains and sustained behavioural change; as behavioural outcomes were secondary and exploratory after a single brief counselling session, these findings should be interpreted cautiously and confirmed in studies with longer follow-up and repeated interventions. Finally, selection bias is possible, as participation required parental presence on the day of surgery and willingness to engage in counselling and follow-up; participating parents may therefore differ from nonparticipants in motivation or health literacy, potentially affecting generalisability. Moreover, as this was a single-centre study conducted in a Belgian tertiary-care hospital, the findings may not be fully generalisable to other healthcare systems or practice settings and should be confirmed in multicentre studies across diverse populations.

Despite these limitations, the study has practical and research implications. Clinically, the peri-operative consultation is a feasible and potentially high-yield moment to deliver brief preventive interventions: parents are present, attentive and often motivated to optimise their child's outcome. Embedding a short, standardised counselling module within the pre-operative or peri-operative workflow could be a low-cost way to increase parental awareness at scale.

To convert awareness into durable behavioural change, the following steps merit evaluation: integrate visual aids and objective feedback (e.g. environmental or biological tests) to make abstract risks tangible; provide take-home materials and digital follow-up (SMS reminders, web resources, apps) to reinforce messages; offer linkage to cessation supports for parents (hotlines, nicotine replacement therapy, behavioural programmes) and tailor messages to parental profile according to education level and prior beliefs, as receptivity varies across subgroups. From a research standpoint, a cluster-randomised trial comparing standard peri-operative care versus structured counselling plus follow-up (with objective exposure endpoints and longer follow-up at 6 to 12 months) would be the logical next step. Such a design would also allow the assessment of downstream clinical endpoints – peri-operative respiratory events in the child, healthcare utilisation and sustained reductions in household exposure. Future studies should evaluate structured peri-operative smoking-reduction pathways using randomised or cluster-randomised designs, objective exposure biomarkers such as cotinine and longer follow-up periods.

Training the peri-operative team is also pivotal. Anaesthesiologists, surgeons and nursing staff can be briefed to raise the topic sensitively and to reinforce messages, but this requires short training modules and workflow adaptations so that prevention becomes a routine part of care rather than an optional add-on.^17^

On a population level, integrating peri-operative counselling into existing tobacco control strategies could multiply the reach of public health campaigns and contribute to reductions in childhood exposure – an achievable and low-cost public health gain.^18^

## Conclusion

A brief, counsellor-led educational intervention delivered in the paediatric peri-operative setting was associated with a prompt and measurable increase in parental awareness about the risks of passive smoking – most notably regarding anaesthetic risk and tertiary smoke. These findings demonstrate the feasibility and acceptability of a brief peri-operative counselling approach but do not establish effectiveness in reducing SHS exposure or improving peri-operative outcomes. However, single-session education alone yielded only modest short-term changes in reported behaviour. Given the single-arm observational design, these findings should be interpreted cautiously and considered hypothesis-generating pending confirmation in adequately powered multicentre randomised studies. These results support the peri-operative consultation as a promising ‘teachable moment’ for tobacco-exposure prevention but underscore the need for multicomponent, sustained approaches and rigorous controlled studies (including objective exposure measures and longer follow-up) to translate awareness into lasting reductions in children's exposure and associated peri-operative risk.

## Supplementary Material

Supplemental Digital Content

## References

[1] Treyster Z Gitterman B. Second hand smoke exposure in children: environmental factors, physiological effects, and interventions within pediatrics. *Rev Environ Health* 2011; 26:187–195. 22206195 10.1515/reveh.2011.026

[2] Farber HJ Groner J Walley S. Protecting children from tobacco, nicotine, and tobacco smoke. *Pediatrics* 2015; 136:e1439–e1467. 26504135 10.1542/peds.2015-3110

[3] Asfaw SM Vijayawada SM Sharifian Y. Protecting young lives: a systematic review of the impact of secondhand smoke exposure and legislative measures on children's health. *Cureus* 2024; 16:e72548. 39606547 10.7759/cureus.72548 PMC11601997

[4] Flor LS Anderson JA Ahmad N. Health effects associated with exposure to secondhand smoke: a Burden of Proof study. *Nat Med* 2024; 30:149–167. 38195750 10.1038/s41591-023-02743-4 PMC10803272

[5] Talluri R Shete SS Shastri SS. Secondhand tobacco smoke exposure in homes and vehicles in youth: disparities among racial, and sexual and gender minorities. *Front Public Health* 2024; 12:1370552. 39109147 10.3389/fpubh.2024.1370552 PMC11300350

[6] Chiswell C Akram Y. Impact of environmental tobacco smoke exposure on anaesthetic and surgical outcomes in children: a systematic review and meta-analysis. *Arch Dis Child* 2017; 102:123–130. 27417307 10.1136/archdischild-2016-310687 PMC5284464

[7] McGrath-Morrow SA Gorzkowski J Groner JA. The effects of nicotine on development. *Pediatrics* 2020; 145:e20191346. 32047098 10.1542/peds.2019-1346 PMC7049940

[8] Zhou S Rosenthal DG Sherman S. Physical, behavioral, and cognitive effects of prenatal tobacco and postnatal secondhand smoke exposure. *Curr Probl Pediatr Adolesc Healthcare* 2014; 44:219–241. 10.1016/j.cppeds.2014.03.007 PMC6876620 25106748

[9] Habre W Disma N Virag K APRICOT Group of the European Society of Anaesthesiology Clinical Trial Network. Incidence of severe critical events in paediatric anaesthesia (APRICOT): a prospective multicentre observational study in 261 hospitals in Europe. *Lancet Respir Med* 2017; 5:e22. 10.1016/S2213-2600(17)30116-9 28363725

[10] Riley C Ladak N. Reducing pediatric exposure to environmental tobacco smoke: The effects of pediatric exposure to environmental tobacco smoke and the role of pediatric peri-operative care. *Paediatr Anaesth* 2020; 30:1199–1203. 32395863 10.1111/pan.13907

[11] Wong J An D Urman RD. Society for Peri-Operative Assessment and Quality Improvement (SPAQI) consensus statement on peri-operative smoking cessation. *Anesth Analg* 2020; 131:955–968. 31764157 10.1213/ANE.0000000000004508

[12] Howard R Englesbe M. Leveraging the peri-operative period to improve population health. *Perioper Med (Lond)* 2023; 12:21. 37277869 10.1186/s13741-023-00311-5 PMC10242805

[13] Myers V Shiloh S Rosen L. Parental perceptions of children's exposure to tobacco smoke: development and validation of a new measure. *BMC Public Health* 2018; 18:1031. 30126404 10.1186/s12889-018-5928-1 PMC6102809

[14] Myers V Shiloh S Zucker DM. Changing exposure perceptions: a randomized controlled trial of an intervention with smoking parents. *Int J Environ Res Public Health* 2020; 17:3349. 32408551 10.3390/ijerph17103349 PMC7277098

[15] Alsahli FA Alruwais NM Alsultan LS. Interventions for prevention of tobacco smoking in school-aged children and adolescents: a systematic review and meta-analysis. *Cureus* 2025; 17:e77008. 39912043 10.7759/cureus.77008 PMC11797488

[16] Pugmire J Sweeting H Moore L. Environmental tobacco smoke exposure among infants, children and young people: now is no time to relax. *Arch Dis Child* 2017; 102:117–118. 28100555 10.1136/archdischild-2016-311652

[17] Flocke SA Albert EL Lewis SA. A cluster randomized trial evaluating a teachable moment communication process for tobacco cessation support. *BMC Fam Pract* 2021; 22:85. 33947346 10.1186/s12875-021-01423-x PMC8097804

[18] Merianos AL Gordon JS Lyons MS. Evaluation of tobacco screening and counseling in a large, midwestern pediatric emergency department. *Tob Prev Cessat* 2021; 7:39. 34056146 10.18332/tpc/134751 PMC8145199

