# Supplementary material for: Impact of a brief peri-operative counselling session on parental awareness of passive smoking in paediatric ambulatory surgery: A single-centre observational study
Source: Eur J Anaesthesiol Intensive Care. 2026 May 22;5(3):1-7. doi: 10.1097/EA9.0000000000000121 (PMC13232896; doi:10.1097/EA9.0000000000000121)
Supplement: Supplemental Digital Content [file ejaic-5-e0121-s001.pdf]

Content of the counselling session

• Description of the different types of tobacco smoke

Primary smoke is the smoke inhaled directly by smokers when their mouth comes into contact with a cigarette, cigar, or other tobacco product. This type of smoke contains more than 4,000 chemical substances, many of them toxic (including about 50 known carcinogens), such as carbon monoxide and tar. Primary smoke, which is inhaled repeatedly, is directly responsible for the harmful health effects observed in smokers, including respiratory diseases, cardiovascular diseases, and cancer.

Second-hand smoke, also referred to as “passively inhaled smoke,” is a mixture of the smoke exhaled by the smoker and the smoke released from the burning end of the tobacco product (e.g. a cigarette smouldering in an ashtray). This smoke results from incomplete, low-temperature combustion of tobacco, which generates an even greater number of chemical substances (up to 7,000, including 69 known carcinogens). Second-hand smoke involuntarily affects non-smokers in the smoker’s surroundings—particularly children and pregnant women—thereby increasing the risk of respiratory diseases, allergies, and other health problems.

Third-hand smoke, also known as “environmental tobacco pollution,” refers to residual tobacco smoke contaminants that accumulate in the environment, including on hair, skin, clothing, furniture, walls, and other surfaces. These residues persist even after the cigarette has been extinguished—sometimes for years—trapped within surfaces, even when the smell has disappeared. Third-hand smoke is found on floors and in household dust. Crawling children and household pets absorb proportionally more third-hand smoke than adults. It can be inhaled or absorbed by individuals who come into contact with contaminated surfaces or enter spaces previously occupied by smokers.

Each of these types of smoke has important health implications. Awareness of their effects is essential to protect both smokers and non-smokers from the hazards associated with tobacco use.

• Definition of second-hand smoke exposure

Second-hand smoke exposure refers to the involuntary inhalation of smoke emitted by one or more smokers. Levels of second-hand and third-hand smoke can be particularly high in enclosed environments such as the home. Smoking in another room, under a kitchen hood, or near an open window is insufficient to eliminate the toxic substances contained in tobacco smoke, which readily spread throughout the environment.

• Health effects of second-hand smoke in children and specific perioperative risks

The concentration of many toxic substances is higher in second-hand smoke than in the smoke inhaled directly by the smoker (pipe and waterpipe/shisha smoking are not less harmful than cigarettes). Children, pregnant women, and individuals with pre-existing health conditions are particularly vulnerable to the harmful effects of passive smoking.

Studies show that second-hand smoke exposure is associated with an increased risk of sudden infant death syndrome, due to the deleterious effects of smoke on the respiratory system. Tobacco smoke also causes irritation of the eyes, nose, and throat. Exposed children are more prone to recurrent upper respiratory tract infections, otitis media, bronchitis, and other respiratory infections. As children grow, passive smoke exposure also worsens symptoms in those with asthma, including an increased risk of asthma attacks.

Exposure to second-hand smoke during childhood is further associated with an increased risk of cardiovascular disease and cancer in adulthood. Children exposed to tobacco smoke may also develop behavioural problems, learning difficulties, and developmental delays.

Second-hand smoke, particularly during the perioperative period, may have several harmful effects on patients:

1. Children exposed to passive smoking may have impaired lung function, increasing the risk of respiratory complications during and after general anaesthesia (e.g. atelectasis or pulmonary infection).
2. Passive smoking can impair blood circulation and tissue healing, potentially delaying surgical wound healing.
3. Smoke exposure may compromise the immune system, increasing the risk of postoperative infections, which is particularly problematic in the surgical context.
4. Patients exposed to second-hand smoke may respond differently to anaesthetic agents, increasing the risk of intraoperative complications and necessitating adjustments to the anaesthetic protocol.
5. Cigarette smoke contains toxic substances that increase oxidative stress in the body, with potentially deleterious effects on multiple organs.
6. The presence of tobacco smoke in the environment may also affect patients' psychological well-being, which can influence postoperative recovery.

Children exposed to second-hand smoke may therefore require closer postoperative monitoring due to their increased vulnerability to complications. Avoiding passive smoking before surgery is crucial to minimise these risks and promote optimal recovery.

• Strategies to reduce second-hand smoke exposure

Practical advice:

- Avoid smoking in the presence of non-smokers, especially children, and in areas accessible to them.
- Opening doors or windows or smoking under a kitchen hood is insufficient to eliminate tobacco smoke. Even if odours are less noticeable, harmful substances remain suspended in the air and continue to spread long after the cigarette has been extinguished. In enclosed spaces, the smoker is also exposed to passive smoke.
- Smoking indoors (including in a separate room) and in vehicles is strongly discouraged to limit second-hand smoke exposure. Smoking should always take place outdoors. Keeping tobacco products near an exit may help initiate a behavioural shift toward smoking “differently” and “elsewhere.”

- Pharmacological and non-pharmacological solutions exist to reduce smoking, decrease consumption, or stop smoking altogether.
- Tobacco specialists (tabacologists) are available to provide information and support.
- Each individual situation is addressed through a personalised approach, respecting the person's decisions, history, and available resources in their effort to become tobacco-free.

For further information or support, we are available at +32 4 323 39 72.

Loïc Le Jeune
Tobacco cessation specialist
llejeune@chuliege.be

Contenu de la sensibilisation :

- **Description des différents courants de fumée** :

La fumée **primaire** est la **fumée inhalée par les fumeurs** lorsque leur bouche entre en contact direct avec une cigarette, un cigare ou un autre produit du tabac. Ce type de fumée contient plus de 4 000 produits chimiques, toxiques (dont 50 sont reconnus comme cancérigènes), dont le monoxyde de carbone et les goudrons. La fumée primaire, fréquemment inhalée, est directement liée aux effets nocifs sur la santé des fumeurs, notamment les maladies respiratoires, les maladies cardiovasculaires et le cancer.

La fumée **secondaire,** également appelée "fumée passivement inhalée", est le mélange de la fumée exhalée par le fumeur, et de la fumée émise par la combustion du tabac (produits qui s'en dégagent). La fumée (un mégot qui se consume dans le cendrier) est le résultat d'une combustion incomplète à faible température du tabac, qui crée plus de substances chimiques (jusqu'à 7000 produits chimiques, dont 69 sont reconnus comme cancérigènes). La fumée secondaire affecte, involontairement l'entourage non-fumeur des fumeurs (en particulier les enfants, les femmes enceintes, augmentant les risques de maladies respiratoires, d'allergies et d'autres problèmes de santé.)

La fumée **tertiaire,** également connue sous le nom de "pollution de l'environnement", la fumée tertiaire fait référence aux résidus de fumée qui s'accumulent dans l'environnement, dans les cheveux, la peau, sur les vêtements, les meubles, les murs et les surfaces. Elle subsiste même lorsque les fumeurs ont éteint leur cigarette, parfois même des années, coincée dans les surfaces, et ce même si l'odeur est disparue. On la retrouve sur le sol, dans la poussière de maison ; Les enfants qui rampent, ainsi que les animaux domestiquent absorbent plus de fumée tertiaire que les adultes. La fumée tertiaire peut être inhalée par des personnes qui entrent en contact avec des surfaces contaminées ou qui se trouvent dans des espaces où des fumeurs ont été.

Chacun de ces types de fumée a des implications pour la santé. Il est essentiel de prendre conscience de leurs effets et de protéger à la fois les fumeurs et les non-fumeurs des dangers associés au tabagisme.

- **Définition du tabagisme passif** :

Il s'agit de l'inhalation involontaire de la fumée dégagée par un ou plusieurs fumeurs.

La quantité de fumée secondaire et tertiaire peut être particulièrement élevée dans un espace clos comme la maison. Fumer dans une autre pièce, sous la hotte ou près d'une fenêtre ouverte ne suffit pas à se débarrasser des substances toxiques qui composent la fumée de tabac. Celle-ci s'infiltre partout.

- **Effets du tabagisme passif sur la santé des enfants et risques spécifiques en chirurgie** :

La concentration de nombreux produits toxiques est plus élevée dans la fumée secondaire que dans celle inhalée directement par le fumeur (la pipe et la chicha ne sont pas moins nocives que la cigarette).

Les enfants, les femmes enceintes et les personnes souffrant de problèmes de santé préexistants sont particulièrement vulnérables aux effets néfastes du tabagisme passif.

Des études montrent que le tabagisme passif est associé à un risque accru de Syndrome de mort subite du nourrisson, en raison des effets nocifs de la fumée sur leur système respiratoire. De plus, la fumée entraîne une irritation des yeux, du nez et de la gorge. L'enfant a tendance à faire davantage de rhinopharyngites, d'otites, de bronchites et d'infections respiratoires. Avec la croissance, la fumée passive augmente également les symptômes des enfants déjà asthmatiques, (y compris le risque de crise d'asthme).

L'exposition au tabagisme passif pendant l'enfance est liée à un risque accru de maladies cardiovasculaires et de cancer à l'âge adulte. Les enfants exposés à la fumée de tabac peuvent également avoir des problèmes de comportement, des troubles d'apprentissage et des retards de développement.

Le tabagisme passif, surtout en période périopératoire, peut avoir plusieurs effets néfastes sur les patients.

1. Les enfants exposés au tabagisme passif peuvent avoir une fonction pulmonaire compromise, ce qui augmente le risque de complications respiratoires pendant et après une anesthésie générale (atélectasie ou l'infection pulmonaire).

2. Le tabagisme passif peut affecter la circulation sanguine et la guérison des tissus, ce qui peut retarder la cicatrisation des plaies chirurgicales.

3. L'exposition à la fumée peut compromettre le système immunitaire, augmentant ainsi le risque d'infections postopératoires, problématiques dans le contexte chirurgical.

4. Les patients exposés au tabagisme passif peuvent avoir des réactions différentes aux anesthésiques, (augmentant le risque de complications durant l'intervention) ce qui peut nécessiter des ajustements dans le protocole anesthésique.

5. La fumée de cigarette contient des substances toxiques qui augmentent le stress oxydatif dans l'organisme, pouvant avoir des effets délétères sur les organes.

6. La présence de fumée de tabac dans l'environnement peut également affecter le bien-être psychologique des patients, ce qui peut influencer leur récupération postopératoire. Les enfants exposés au tabagisme passif peuvent ainsi nécessiter un suivi postopératoire plus attentif en raison de leur vulnérabilité accrue face à des complications.

Éviter le tabagisme passif avant une intervention chirurgicale est donc crucial pour minimiser ces risques et favoriser une récupération optimale.

- **Modalités de réduction du tabagisme passif** :

Conseils pratiques :

- Eviter de fumer en présence de non–fumeurs et particulièrement d'enfants et dans les locaux auxquels ces derniers ont accès.
- Il ne suffit pas d'ouvrir portes et fenêtres ou de fumer sous la hotte pour faire disparaître la fumée. Même si on sent moins les odeurs, les substances nocives restent en suspension dans l'air. La fumée continue à se répandre et persiste longtemps après que la cigarette soit écrasée. De plus, en milieu clos, le fumeur est lui aussi victime du tabagisme passif.
- Fumer à l'intérieur du domicile (y compris dans une pièce), et dans son véhicule, est fortement déconseillé pour limiter le tabagisme passif. Il est, dès lors, conseillé de toujours fumer à l'extérieur. Disposer le tabac dans un endroit proche de l'extérieur, afin de débuter une démarche active de fumer “autrement”, “ailleurs”.
- Des solutions (médicamenteuses et non médicamenteuses) existent pour limiter le tabagisme, diminuer ou cesser de fumer.
- En cas de questions, des informations sont disponibles avec les tabacologues.
- Chaque situation particulière est abordée suivant une solution personnalisée, en accord avec la décision, l'histoire et les ressources de chaque personne désirant se libérer du tabac.
- Nous sommes disponibles au 04/323.3972

Loïc Le Jeune,
Tabacologue
llejeune@chuliege.be
